# Bacterium-Like Particles for Efficient Immune Stimulation of Existing Vaccines and New Subunit Vaccines in Mucosal Applications

**DOI:** 10.3389/fimmu.2013.00282

**Published:** 2013-09-17

**Authors:** Natalija Van Braeckel-Budimir, Bert Jan Haijema, Kees Leenhouts

**Affiliations:** ^1^Mucosis BV, Groningen, Netherlands

**Keywords:** carrier-adjuvant, mucosal vaccine technology, particles, *Lactococcus lactis*, influenza vaccines, RSV vaccines

## Abstract

The successful development of a mucosal vaccine depends critically on the use of a safe and effective immunostimulant and/or carrier system. This review describes the effectiveness and mode of action of an immunostimulating particle, derived from bacteria, used in mucosal subunit vaccines. The non-living particles, designated bacterium-like particles are based on the food-grade bacterium *Lactococcus lactis*. The focus of the overview is on the development of intranasal BLP-based vaccines to prevent diseases caused by influenza and respiratory syncytial virus, and includes a selection of Phase I clinical data for the intranasal FluGEM vaccine.

## Introduction

A key issue in the development of improved and new vaccines is safety, since most vaccines are given to healthy individuals. In order to improve safety profiles, the use of well-defined (recombinant) purified antigens for the generation of subunit vaccines has become key in vaccine development programs. In addition, there is an increasing interest to explore other modes of vaccine administration besides the use of needles. Since purified soluble antigens are usually poorly immunogenic, even more so when delivered through mucosal (nasal, oral) routes, the addition of safe immunostimulators/adjuvants to increase the efficacy of vaccines is needed (1–3).

**Figure 1:**
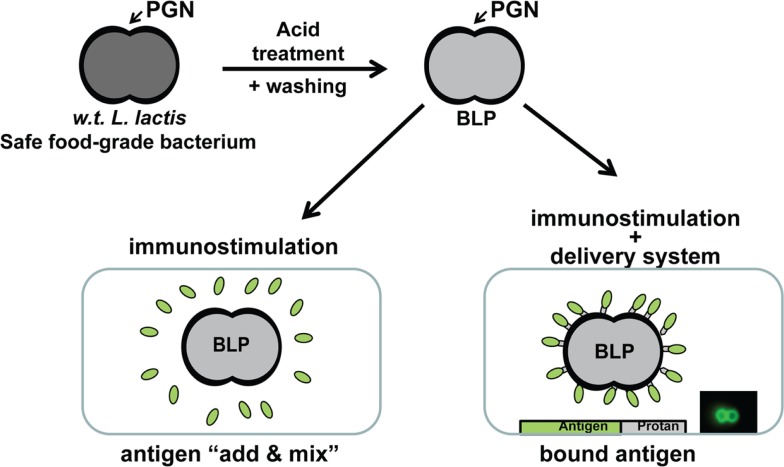
**Overview of the production and use of BLPs**. After treatment in hot acid, degradation products and acid are removed by washing with phosphate buffered saline (PBS). The BLPs are finally formulated in PBS. Vaccines are made by BLPs admixed with antigens (this formulation is of particular interest for the reformulation of existing vaccines) or antigens are bound to the surface of the BLPs. For this latter format, it is a requirement that the subunit antigens are produced as a fusion protein with the Protan tag in a suitable production organism. Mixing of an antigen-Protan solution with BLPs results in instant, strong, and stable non-covalent binding such that BLPs are completely covered at the surface with the antigen.

In order to minimize regulatory hurdles, we have developed a non-living particle that can be used as an immunostimulant for the improvement of existing vaccines and to enable mucosal application, or can be used additionally as a vaccine carrier for subunit antigens. The particles are based on non-recombinant *Lactococcus lactis* bacteria. *L. lactis* is an innocuous Gram-positive bacterium that is commonly used in the food industry; it has Generally Recognized As Safe (GRAS) status of the Food and Drug Administration (FDA). The safe background of *L. lactis* makes it highly suitable for use in vaccines. The Gram-positive bacterial cell surface consists of a single membrane on the inside and a thick cell-wall on the outside (4, 5). The cell-wall is built up of multiple layers of peptidoglycan (PGN) with various other components that may protrude both on the inside and outside. A simple pretreatment in hot acid destroys all cellular components, including intracellular components such as DNA. Cell-wall components other than the rigid PGN matrix are also degraded. The result is a non-living particle that retains the same shape and size as the bacterium before treatment. Acid treatment is followed by extensive washing with buffer to remove acid and degradation products (6). The procedure results in non-living spherical shaped bacterium-like particles (BLPs) that have a diameter of approximately 1–2 μm and consist predominantly of a PGN outer surface (Figure 1).

As previously mentioned, BLPs are used in two different formats. They are used as an immunostimulant by simply mixing with vaccine antigens (admixed). This format is of particular interest in the reformulation of existing vaccines to enable mucosal application. The preferred format for use in recombinant subunit vaccines is a formulation in which the antigens are bound to the surface of BLPs. Binding of antigens to BLPs requires the presence of a PGN binding tag (Protan) in the antigen. The PGN binding domain of the *L. lactis* AcmA cell-wall hydrolase (7) has been used for this purpose (8). Antigen-Protan fusions have been produced in prokaryotic (*L. lactis*, *E. coli*) and eukaryotic hosts (insect, CHO, and HEK cells). To date, over 40 different antigens of bacterial, viral, or parasitic nature have been successfully overexpressed as Protan fusions using these expression hosts (9). The Protan fusions are secreted preferably by the expression cells, allowing easy removal of the production cells. Conventional protein isolation techniques are then used to purify the Protan fusion product. The purified fusion is subsequently mixed with BLPs to allow binding. The BLPs with bound antigen-Protan fusion are subsequently recovered, washed, and formulated in a suitable buffer (Figure 1).

A more extensive overview of BLP and Protan characteristics was recently published elsewhere (9).

## Immunogenicity of BLP-Based Vaccines: *In vivo* Studies

Although the focus of this paper is on the mucosal use of BLP-based vaccines, Table 1 provides an overview of all mucosal and parenteral BLP-based vaccines tested to date as a proof-of-concept for immunogenicity and protection against pathogenic challenge. The immunogenicity and protection capacity of both BLP-based admixed vaccines and vaccines in which the antigen was bound to the BLPs has been tested extensively in various animal models. The listed studies demonstrate robust antigen-specific systemic immune responses after parenteral vaccination and both strong local and systemic responses induced upon mucosal vaccination. Furthermore, the induced immune responses have proven to be protective against infections with specific pathogens, including viruses, bacteria, and parasites.

**Table 1:** **Overview of preclinical proof-of-concept studies performed using different BLP-based vaccine formulations**.

Pathogen		Antigenformulation	Vaccinationroute	Animalmodel	Testedparameter	Study outcome
**IMMUNOGENICITY AND PROTECTION CAPACITY OF BLP-BASED VACCINES**						
**Virus**	Influenza	Subunit vaccine mixed with BLPs	i.m.	Mouse, rat, rabbit, ferret	Correlate of protection	Serum HI titers >40 with strong increase compared to benchmark^a^ (10)
				Mouse	i.n. Homologous challenge	100% Protection, inhibition of viral replication in the lungs (10)
			i.n.	Mouse, rat, rabbit, ferret	Correlate of protection	Serum HI titers >40 comparable to i.m. benchmark^a^ (11)
		Split-virion vaccine mixed with BLPs	i.n.	Mouse	i.n. Homologous challenge	100% Survival with strong reduction of lung viral load (12)
				Mouse	i.n. heterologous challenge	100% Protection with strong reduction of lung viral load, superior compared to benchmark (12)
				Mouse	Local mucosal response	S-IgA titers in the lung, nose and vaginal mucosa (11, 12)
		Subunit vaccine mixed with BLPs	i.g.	Mouse	Correlate of protection	Serum HI titers >40 (13)
				Mouse	Local mucosal response	S-IgA titers in intestinal and nasal lavages (13)
		HA bound to BLPs	i.m.	Mouse	Correlate of protection	Serum HI titers >40, strong increase compared to i.m. benchmark^b^
			i.n.	Mouse	Correlate of protection	Serum HI titers >40 comparable to i.m. benchmark^b^
		M2e bound to BLPs	i.n.	Mouse	i.n. challenge	100% protection, strong induction of lung viral load^c^
		NP bound to BLPs	i.n.	Mouse	Cellular response	Th1/Th2 balanced cellular response (IFNγ/IL4 ratio)^c^
	RSV	RSV F bound to BLPs	i.n.	Mouse, cotton rat	Correlate of protection	Virus neutralization titers measured in serum (38)
				Mouse, cotton rat	i.n. challenge	Strong reduction in lung virus titers (38)
				Mouse	Local mucosal response	S-IgA titers in nasal washes (38)
				Cotton rat	Safety	Absence of enhanced disease symptoms (interstitial pneumonia, alveolitis) (38)
	HBV	HBsAg mixed with BLPs	i.n.	Mouse, rat	Correlate of protection	Serum titers >10 U/ml, comparable to i.m. benchmark^c^
**Bacteria**	*Streptococcus pneumoniae*	IgA1p, SlrA, PpmA bound and mixed to BLPs	i.n., i.m.	Mouse	Pulmonary challenge (pneumonia model)	50–75% Protection associated with strong reduction in bacteremia (14)
					i.n. Challenge (colonization model)	Strong reduction in nasopharyngeal colonization^c^
	*Yersinia pestis*	LcrV bound to BLPs	i.n.	Mouse	i.v. Challenge	100% Protection (15)
			i.g.	Mouse	i.v. Challenge	Up to 85% protection^d^
	*Shigella* spp.	IpaB, IpaD bound to BLPs	i.n.	Mouse	Pulmonary challenge	100% Protection against *S. flexneri* in adults; partial protection against *S. flexneri* in newborns; 90% cross-protection against *S. sonnei*^d^
**Parasites**	*Plasmodium berghei*	CSP bound to BLPs	i.m.	Mouse	Infected mosquito challenge	100% Protection; sterile immunity (16)

RSV, respiratory syncytial virus; HBV, hepatitis B virus; i.m., intramuscular; i.n., intranasal; i.g., intragastric; i.v., intravenous; HI, hemagglutination inhibition; S-IgA, secretory IgA; HA, hemagglutinin; M2e, M2 ectodomain; NP, nucleoprotein; F, fusion protein; HBsAg, HBV surface antigen; IgA1p, immunoglobulin A1 protease; SlrA, streptococcal lipoprotein rotamase A; PpmA, proteinase maturation protein A; LcrV, low-calcium response virulence antigen; Ipa, invasion plasmid antigen; CSP, circumsporozoite protein; ^a^seasonal i.m. non-adjuvanted influenza subunit or split-virion vaccine; ^b^manuscripts in preparation; ^c^mucosis, unpublished data; ^d^Pasetti, unpublished data.

### Mucosal administration of BLP-based vaccines against respiratory viruses

Most intensively studied and advanced BLP-based vaccines are formulations against respiratory viruses including: seasonal influenza vaccine (FluGEM) and Respiratory Syncytial Virus (RSV) vaccine (SynGEM). In the following section we provide an overview of the results of immunization and protection studies performed in influenza- and RSV-animal models.

#### FluGEM in animal models

Influenza is an acute respiratory illness that mostly affects the upper, and sometimes also the lower, respiratory tract and is caused by the influenza virus. It represents an important, often underestimated public health problem and is associated with increased general practice consultation rate, hospital admissions and excess deaths (17). In addition, influenza has a high impact on health care planning, and is also one of the major causes of increased absenteeism from work and school, and thus has significant economic impact. Every year approximately 5–10% of the global population is infected with influenza, while during a major epidemic the attack rate might increase up to 50%. The World Health Organization (WHO) has estimated that 3–5 million of annually infected people develop a severe form of the disease and of those, 250,000–500,000 die (18).

Influenza virus is a negative-sense ssRNA virus and it belongs to the family of *Orthomyxoviridae* (19). The virion is composed of an external envelope derived from plasma membrane of the infected cell that contains viral surface glycoproteins. Additionally, the viral particle contains an internal core, composed of the viral genome associated with specific proteins (20). The surface of the viral particle is covered by numerous protein spike-like projections. These are molecules of hemagglutinin (HA) and neuraminidase (NA); two major surface glycoproteins (20). Besides HA and NA, the viral envelope also contains membrane protein 2 (M2) (20). In addition to the envelope glycoproteins, the genome of influenza virus also encodes the matrix protein (M1), viral polymerase proteins, the nucleoprotein (NP), and a number of non-structural proteins (20).

Vaccination is the most effective method of preventing influenza virus infection and its potentially severe complications. Current influenza vaccination strategies are mostly based on inactivated virus vaccines (subunit, split-virion, virosome, whole inactivated virus), which are generally administered through intramuscular injection and induce antibodies against HA – one of the two surface viral glycoproteins and the main antigenic component of the virus (21). Parenterally administered vaccines usually induce potent systemic responses, but no local, mucosal response at the port of viral entry. This lack of induced mucosal response might be a limitation of the protective capacity of such vaccines (22).

Our goal is the development of a mucosal, more specifically, an intranasal influenza vaccine. In contrast to parenteral vaccination, this route of vaccine administration would activate local mucosal responses at the port of viral entry, e.g., secretory IgA (S-IgA), in addition to systemic responses. Therefore, an intranasal vaccine may provide a powerful first line of defense against influenza. This would lead to reduction of virus entry, but also to reduction in virus replication and shedding. Furthermore, intranasal vaccination does not require trained health care personnel for the administration of the vaccine and does not bear the risk of needle stick injuries (23).

In order to achieve maximum immunogenicity when introduced through the i.n. route, inactivated influenza vaccines require the presence of immunostimulating compounds. Several adjuvants have been extensively evaluated as potential candidates for mucosal vaccination, such as nucleic acids and bacterial components (e.g., toxins) (24, 25). Despite good immunogenicity profiles, development of many of the aforementioned adjuvants is hampered by safety and regulatory concerns (26). Therefore, a safe adjuvant/immunostimulant, suitable for i.n. influenza vaccination, with a good immunopotentiating capacity is still highly desirable.

We have developed two types of intranasal influenza vaccines. The first type, FluGEM-A (A stands for admixing), is based on a mixture of BLPs with commercially available influenza vaccine antigen (subunit or split-virion vaccine). The second type, FluGEM-B (B stands for bound), contains purified influenza HA and/or M2 protein ectodomain (M2e) bound to BLPs, as described in the Section “Introduction.” Both vaccines have been extensively tested for safety and immunogenicity in animals, and FluGEM-A has also been tested in a Phase I clinical trial (see Final Remarks of this review).

##### Influenza antigen admixed with BLPs: FluGEM-A

To evaluate the immunogenicity of FluGEM-A, mice were vaccinated i.n. with FluGEM-A formulation, composed of seasonal subunit vaccine mixed with BLPs. Control groups were i.m. (benchmark) or i.n. vaccinated with a non-adjuvanted equivalent.

##### Immunogenicity of FluGEM-A vaccine

**Figure 2:**
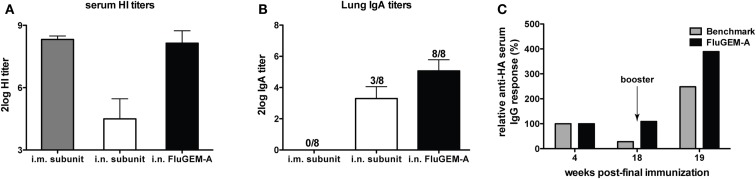
**Magnitude and duration of FluGEM-A – induced immune response**. Groups of eight mice were vaccinated three times (day 0, 14, and 28) i.n. with FluGEM-A or with benchmark subunit vaccine administered through i.n. or i.m. route. One vaccination dose contained 5 μg HA and in addition to antigen, FluGEM-A vaccine contained 0.3 mg BLPs. HI titers **(A)** measured in the sera of mice i.n. vaccinated with FluGEM-A were comparable to titers induced upon i.m. vaccination with benchmark subunit vaccine, and higher in comparison to titers induced by i.n. administration with subunit vaccine. S-IgA titers in lung washes **(B)** were measured in all the mice vaccinated i.n. with FluGEM-A, while only three mice from i.n. subunit group and none of the mice from i.m. subunit group had detectable lung S-IgA titers. Serum IgG titers **(C)** induced by i.n. vaccination with FluGEM-A remained stable throughout the 18-weeks post-immunization follow-up period and were still boostable.

Intranasal vaccination of mice with FluGEM-A induced serum hemagglutination inhibition (HI) titers well above the determined protective titer of 40, which was comparable with HI titers measured in the sera of animals vaccinated i.m. with seasonal subunit (benchmark) vaccine, and significantly higher than titers induced by i.n. vaccination with unadjuvanted subunit vaccine (11) (Figure 2A). Similarly, influenza HA-specific IgG titers comparable to those induced upon i.m. benchmark vaccination were measured in sera of mice i.n. vaccinated with FluGEM-A (11). Importantly, subtyping of influenza HA-specific IgG antibodies revealed that i.n. vaccination with FluGEM-A induced well-balanced responses, with an IgG2a/IgG1 ratio of 0.9. Cytokine profiles assessed in spleens of vaccinated animals were characterized by higher production of Th1-polarized cytokines (IFN-γ and IL-2) and lower production of IL-4, a Th2-polarized cytokine, when compared to cytokines induced upon benchmark vaccination (11). Finally, i.n. immunization with FluGEM-A, in contrast to benchmark vaccination, induced strong local S-IgA responses, especially important as the first line of defense at the port of virus entry (11, 12) (Figure 2B).

In addition to strong systemic and mucosal responses, i.n. vaccination with FluGEM-A induced immune responses that seem to wane slower than the response induced upon i.m. vaccination with a non-adjuvanted benchmark vaccine. As depicted in Figure 2C, serum IgG titers induced by i.n. vaccination with FluGEM-A remained stable throughout the follow-up period of almost 4 months after the final immunization and were still boostable. This observation is suggestive of induction of influenza-specific memory B-cells and the presence of long-lived antibody secreting plasma cells. On the other hand, IgG titers induced by i.m. benchmark vaccination after 4 months decreased to approximately a quarter of the initial post-vaccination value.

Similar immunogenicity profiles and immunopotentiating capacities of BLPs were demonstrated for FluGEM-A in other animal models such as rats, rabbits, and ferrets (9).

##### Protection against homologous and heterologous challenge induced by intranasal vaccination with FluGEM-A

**Figure 3:**
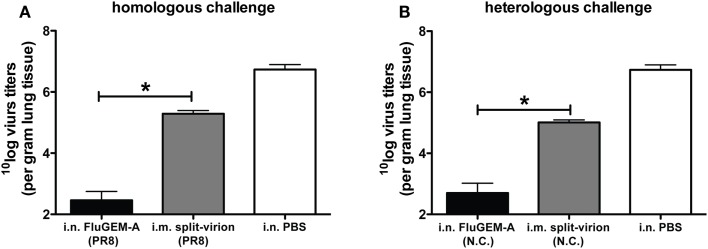
**Virus titers measured in the lungs of mice exposed to homologous and heterologous influenza challenge upon i.n. vaccination with FluGEM-A**. Groups of six mice were vaccinated three times (day 0, 14, and 28) i.n. with FluGEM-A or i.m. with benchmark split-virion vaccine. Two groups were vaccinated with vaccine derived from PR8 strain **(A)**, and two groups were vaccinated with vaccines derived from New Caledonia strain **(B)**. One vaccination dose contained 5 μg HA and in addition to antigen, FluGEM-A vaccine contained 0.3 mg BLPs. Three weeks after the final immunization (day 49) mice were exposed to challenge with 100 TCID_50_ of PR8 virus. Lung virus titers were determined 5 days post-challenge. Virus titers measured after homologous challenge in the lungs of mice vaccinated i.n. with PR8-derived FluGEM-A vaccine were up to 100-fold lower compared to titers measured in lungs of mice vaccinated i.m. with PR8-derived split-virion vaccine **(A)**. Virus titers measured after heterologous challenge in the lungs of mice vaccinated i.m. with New Caledonia-derived split-virion vaccine were significantly higher than titers measured in the lungs of mice vaccinated i.n. with New Caledonia-derived FluGEM-A vaccine (B). *p < 0.05; one-tailed Mann–Whitney *U* test (*n* = 6).

The protection capacity of i.n. administered FluGEM-A vaccines was assessed in homologous challenge studies using an influenza PR8 challenge model. To this end, mice were two or three times i.n. vaccinated with FluGEM-A containing a PR8-derived split-virion vaccine (12). Control mice were vaccinated i.m. or i.n. with non-adjuvanted split-virion vaccine. Results of the study clearly demonstrated that vaccination with two or three doses of FluGEM-A vaccine confers solid protection against infection with a homologous virus strain. The degree of protection (measured as loss of body weight after challenge) was comparable to protection observed in the group vaccinated i.m. with the benchmark split-virion vaccine. In contrast, i.n. vaccination with the non-adjuvanted split-virion vaccine-induced only marginal protection, observed in only 20% of vaccinated animals, which provides direct evidence of the mucosal immunostimulating properties of BLPs. Interestingly, although the observed degree of protection between mice vaccinated i.n. with FluGEM-A and those vaccinated i.m. with split-virion vaccine was similar, the measured lung virus titers differed significantly between these two groups. More specifically, in the lungs of mice vaccinated i.n. with FluGEM-A up to 100-fold lower viral load was measured compared to lungs of mice i.m. vaccinated with split-virion vaccine (Figure 3A). This is an important observation, as it suggests that i.n. vaccination with FluGEM-A might reduce the shedding of the virus by infected individuals, and thus control the infection at the population level.

The protection level of an i.n. FluGEM-A vaccine against infection with a heterologous influenza virus was also tested (12). For that purpose, mice vaccinated three times i.n. with FluGEM-A containing influenza A/New Caledonia-derived split-virion vaccine were challenged with PR8 virus. The control group was i.m. vaccinated with a A/New Caledonia-derived split-virion vaccine. Complete protection was observed only in mice vaccinated with i.n. FluGEM-A vaccine, while a partial protection was observed in animals i.m. vaccinated with the split-virion vaccine. This was also reflected in the lung viral load, which was significantly higher in benchmark vaccinated mice (Figure 3B). One possible explanation for the better clearance of the virus upon both homologous and heterologous influenza challenge in mice i.n. vaccinated with FluGEM-A could be the presence of S-IgA antibodies at the mucosal surfaces of vaccinated animals. It has been shown by others that mucosal vaccination against influenza infection induces full protection, which was dependent on the presence and abundance of mucosal S-IgA antibodies (27, 28).

Additional mucosal vaccination routes for delivery of FluGEM-A were also explored. A good example of successful mucosal vaccination with influenza subunit vaccine mixed with BLPs is vaccination through the intragastric (i.g.) route (13). Intragastric vaccination of mice with three doses of FluGEM-A containing a seasonal influenza subunit vaccine, induced HI titers above the established protective criterion (>40). High serum IgG and local nasal and intestinal S-IgA titers were detectable 3 weeks after the last vaccination. Similarly to i.n. vaccination, i.g. vaccination with FluGEM-A induced a balanced IgG2a/IgG1 response.

These summarized data clearly demonstrate the immune-stimulating properties of BLPs and the ability to convert conventional influenza vaccines into effective mucosal vaccines. Importantly, the immune responses elicited by FluGEM-A provide solid protection against both homologous and heterologous influenza infections, and this protection seems to be superior to the protection observed after i.m. benchmark vaccination. Finally, mucosal vaccination with FluGEM-A, but not i.m. benchmark vaccination, has the capacity to induce robust local S-IgA responses at viral port of entry, which may contribute to superior lung protection after infection and reduced viral shedding.

##### Influenza antigen bound to BLPs: FluGEM-B

**Figure 4:**
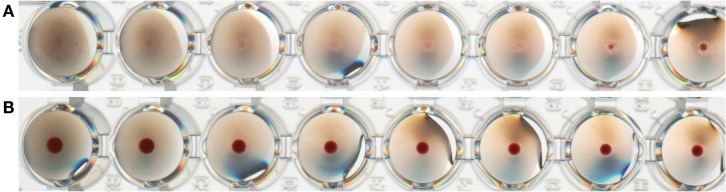
**Biological activity of trimeric and monomeric HA-Protan fusion protein bound to BLPs (FluGEM-B) expressed as hemaggluti nation capacity (HAU)**. Trimeric HA bound to BLPs displays a high capacity to agglutinate turkey red blood cells **(A)**, while monomeric HA bound to BLPs displays no hemagglutination property **(B)**. This suggests that trimeric HA bound to BLPs is properly folded and in biologically active conformation.

Another type of BLP-based influenza vaccine is prepared by physical coupling (non-covalent binding) of purified influenza antigen (e.g., HA, M2e, NP) to the surface of BLPs, as described in the Section “Introduction” of this review. The main aim of this approach is to also use the BLP as a carrier that present the antigens in a biologically active manner to the immune system. To this end, trimeric HA influenza proteins with a Protan tag were produced in mammalian cell expression systems. The HA-Protan fusion protein is able to form trimers because of the presence of a synthetic multimerization domain that replaces the native HA transmembrane and cytoplasmic domains. The correctly folded trimeric HA-Protan fusion protein is purified from the production medium and as such bound to the surface of BLP (FluGEM-B). FluGEM-B with trimeric HA is biologically active as demonstrated by its ability to agglutinate red blood cells to a high extent (Figure 4A), in contrast to its monomeric counterpart bound to BLPs, which does not display any hemagglutination properties (Figure 4B).

There are a few potential advantages of the FluGEM-B concept over FluGEM-A. An important safety aspect is that the process of FluGEM-B preparation allows for simple purification of the recombinant HA protein from the host cells and does not require material originating from (inactivated) viruses, which is required for the preparation of FluGEM-A. Furthermore, the FluGEM-B concept allows for inclusion of purified influenza (and non-influenza) antigens other than HA. This would be particularly beneficial for preparation of multivalent vaccines, e.g., influenza pandemic vaccine based on combination of HA and more conserved proteins (M2e, NP). Finally, it has been shown that physical coupling of soluble (recombinant) antigens to an adjuvant enhances vaccine immunogenicity, by allowing the antigen to be delivered to the same cell activated by the adjuvant (29, 30).

**Figure 5:**
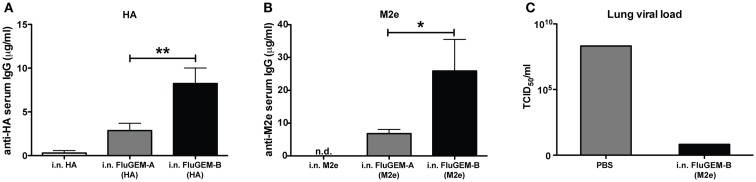
**Immune responses induced upon i.n. vaccination with FluGEM-A and FluGEM-B**. Groups of 10 mice were vaccinated i.n. with HA- or M2e-based FluGEM-A or FluGEM-B. Vaccination dose in the case of HA-based vaccines **(A)** was 1 μg HA mixed with or bound to 0.3 mg BLP. Animals received in total three doses (day 0, 10, and 20) and were sacrificed 2 weeks after the final immunization (day 34). Vaccination dose in the case of M2e-based vaccines **(B)** was 6 μg M2e equivalent mixed with or bound to 0.38 mg BLPs. Animals received in total three doses (day 0, 21, and 42) and were sacrificed 3 weeks after the final immunization (day 63). In both cases physical coupling of the antigen (FluGEM-B) induced a significant increase in serum IgG titers. *p < 0.05; **0.01; one-tailed Mann–Whitney *U* test (*n* = 10). **(C)** Mice (*n* = 3) were vaccinated three times (day 0, 21, and 42) i.n. with M2e-based FluGEM-B vaccine containing 50 μg M2e and 0.3 mg BLPs. Three weeks after the final immunization mice were exposed to challenge with 4LD_50_ × 47 (H3N2) influenza virus. Animals were sacrificed 6 days post-challenge and virus titers were evaluated as a protection parameter. In lungs of all infected mice vaccinated i.n. with M2e-based FluGEM-B decrease of viral load was observed, which indicates protection capacity of the FluGEM-B vaccine.

To probe the difference between FluGEM-A and FluGEM-B regarding immunogenicity, we vaccinated mice i.n. with both vaccine formulations, using influenza HA and M2e as antigens. Control groups were i.n. vaccinated with the antigen only. Figure 5 depicts the results of the studies expressed as vaccine-induced influenza HA- (Figure 5A) or M2e-specific (Figure 5B) serum IgG antibody titers. In both studies, only vaccination with FluGEM-B formulations induced seroconversion in all the animals, while vaccination with antigen (HA or M2e) alone induced low serum IgG titers in only a few mice. Moreover, physical coupling of BLP with the antigens contributed to further, approximately fourfold, increase in IgG titers. Additionally, i.n. vaccination with M2e-based (Figure 5C) and HA-based (to be published elsewhere) FluGEM-B induced protection against virus challenge in vaccinated mice, as demonstrated by the decrease in lung viral load in infected vaccinated animals.

Binding of antigen to BLPs presents the further refinement of BLP-based vaccines, as it allows for easier preparation of highly purified proteins with well-preserved biological activity. This concept might also be beneficial for the preparation of multivalent vaccines, as it allows for simple incorporation of different viral antigens in the same vaccine formulation. Finally, it corroborates the assumption that subunit antigens require physical interaction with the adjuvant for optimal vaccine immunogenicity, as illustrated by the examples of HA- and M2e-based vaccines.

#### Respiratory syncytial virus vaccine: BLP-RSV F (SynGEM)

Respiratory syncytial virus represents a very important target for development of successful vaccine candidates, as it is the single most important cause of viral bronchiolitis in infants and young children and it is the world-wide leading cause of infant hospitalization (31). Furthermore, elderly and immune-compromised individuals are also at high risk of developing severe RSV disease (32, 33). Currently, there is no vaccine available and the only commercially available prophylactic treatment is based on the neutralizing antibody Palivizumab (34, 35). Although there is a clear unmet medical need, the development of a safe RSV vaccine has been hampered for decades by the fiasco of the first RSV vaccine developed, Formalin-inactivated RSV (FI-RSV) which induced exacerbation of disease symptoms in a high percentage of vaccinated children and increased the rate of hospitalizations among vaccinees, including some fatalities (36).

With respect to a candidate RSV vaccine, we are developing an intranasal formulation, SynGEM, that is based on the RSV fusion protein (RSV F) bound to BLPs. The choice of antigen was based on the finding that RSV F protein is highly conserved throughout different virus isolates (37) and the only available neutralizing antibody Palivizumab is directed against this antigen. The F antigen in SynGEM was produced as a functional trimeric prefusion F protein in a similar way as trimeric HA was produced. The immunogenicity of SynGEM has been tested in a mouse and cotton rat model. Interestingly, results of both mouse and cotton rat immunization studies with SynGEM were well in line with immunogenicity results obtained from immunizations of mice with FluGEM (11). More specifically, i.n. vaccination with SynGEM induced robust systemic IgG and local S-IgA titers, with a balanced IgG2a/IgG1 ratio. In contrast, i.m. vaccination with FI-RSV induced no local S-IgA and a very low IgG2a/IgG1 ratio (<0.1), indicating an extremely Th2-polarized response (38). Additionally, unlike i.m. vaccination with FI-RSV, mucosal vaccination with SynGEM induced protective virus neutralizing titers, which represent the accepted correlate of protection against RSV. Finally, i.n. vaccination with SynGEM induced a similarly balanced cytokine profile (IFN-γ/IL4 ratio), as observed upon i.n. vaccination with FluGEM (11).

One of the most important criteria for a successful RSV vaccine is safety in the context of absence of induction of enhanced disease symptoms. It has been suggested that increased production of IL4 is responsible for exacerbation of disease symptoms, as manifested by severe eosinophilia, lack of neutralizing antibodies and lack of efficient cellular responses (e.g., cytotoxic T lymphocytes – CTLs) (39, 40). Therefore, vaccination-induced IL4 levels are used as a predictor of the potential immunopathology. An illustrative example of severely Th2 skewed immune responses induced by vaccination is immunization with alum-adjuvanted FI-RSV (41). In contrast, i.n. vaccination with SynGEM induces very low levels of IL4 and the cytokine profile is dominated by production of IFN-γ, results that are clearly in line with those obtained with i.n. FluGEM vaccination. Additionally, as expected based on the cytokine profile, histopathological analyses in cotton rats indicated that i.n. vaccination with SynGEM does not induce symptoms of enhanced disease (38).

In summary, intranasal vaccination with SynGEM vaccine that is based on trimeric RSV prefusion F protein bound to BLPs, induces robust, well-balanced systemic and local humoral responses. The induced responses were shown to be protective against RSV infection, without inducing enhanced disease symptoms. Therefore, SynGEM represents a promising and safe RSV vaccine candidate.

## Mode of Action of BLPs

### Activation of innate immune system

In Section “Immunogenicity of BLP-Based Vaccines: *In vivo* Studies” of this overview, examples of different vaccine formulations (FluGEM-A, FluGEM-B, SynGEM) were shown, in which mucosal BLP-based vaccines induce robust, long lasting adaptive immune responses. Successful activation of specific adaptive immune responses relies on optimal activation of innate immune responses, which controls and determines the development of specific adaptive response (42, 43). Dendritic cells (DCs) play a crucial role in this process, by being the direct link between the innate and adaptive responses (44). Activation of DCs typically results in cytokine and chemokine production, which further on determine the nature of cellular and humoral responses (45). Finally, only mature and activated DCs have the capacity to (cross-) present the target antigen to CD4+ and CD8+ T-cells (46). The capacity of BLPs to stimulate the innate immunity was well demonstrated in the study of Ramirez et al. (15). *In vitro* stimulation of murine and human neonatal and adult DCs with BLPs induced maturation of these cells, as illustrated by the increase in expression of surface maturation markers (CD40, CD80, CD86, and MHC class I for murine DCs; CD80, CD83, CD86, and HLA-DR for human DCs). These results are summarized in Table 2. Upon acquisition of a mature phenotype, DCs become capable of antigen (cross-) presentation, which activates the cellular arm of the adaptive immune response. In line with this dogma is the observation that DCs stimulated with BLPs display reduced capacity for antigen uptake, which indicates that these cells became effective antigen-presenters (15).

**Table 2:** **BLP-induced maturation and activation of mouse and human DCs**.

	BLP				LPS^m^ or TNFα^h^			
	Neonatal		Adult		Neonatal		Adult	
	Mouse	Human	Mouse	Human	Mouse	Human	Mouse	Human
**BLPs INDUCE MATURATION AND ACTIVATION OF MOUSE AND HUMAN DCs**								
**Markers (fold increase relative to non-stimulated control)**								
CD40^m^ or CD83^h^	1.0	2.2	1.3	3.6	1.0	2.9	1.3	3.0
CD80	1.2	2.2	1.1	2.5	1.4	2.7	1.8	1.8
CD86	4.0	5.1	7.2	6.9	4.2	4.8	6.1	5.1
I-Ad^m^ or HLA-DR^h^	1.1	1.4	8.9	3.1	0.9	1.4	7.2	3.2
**Cytokines (fold increase relative to non-stimulated control)**								
IL-12p70	1.7	2.3	1.7	1.6	2.1	1.5	1.6	2.0
TNFα	140.1	163.0	9.4	541.8	93.4	24.4	10.8	1119.7
IL-10	3.6	4.1	11.4	47.6	3.6	16.3	20.3	2.1
IL6	565.5	164.7	421.3	329.1	707.9	1108.3	428.6	1.6
IFNγ^m^ or IL-1β^h^	1.5	1.7	3.7	3.1	1.9	2.3	7.3	2.0
MCP1^m^ or IL8^h^	1.4	58.9	1.7	13.4	2.9	125.5	1.7	2.6

Maturation and activation of neonatal and adult DCs were evaluated by measuring the upregulation of surface markers and production of specific cytokines. ^m^, mouse; ^h^, human.

In addition to the induction of a mature phenotype, BLPs stimulated these DCs to produce proinflammatory, Th1-promoting and regulatory cytokines. Cytokines such as IL-12p70, TNF-α, IL-10, IL-6, and IFN-γ were secreted by DCs as a response to stimulation with BLPs (Table 2).

Importantly, DCs that have acquired a mature and activated phenotype, as a consequence of stimulation by BLPs, are capable of presenting a specific antigen to target CD4+ and CD8+ T-cells (15). In the study of Ramirez et al. the *Y. pestis* LcrV model antigen, was bound to BLPs (BLP-LcrV) and used for *in vitro* activation of specific CD4+ and CD8+ T-cells. DCs stimulated with such a BLP-associated antigen successfully (cross-) presented the antigen to target cells, as illustrated by remarkable proliferation and production of IFNγ. Additionally, intranasal immunization with BLP-LcrV induced strong activation of antigen-specific antibody secreting cells in the NALT, bone marrow and spleen. The latter organs are considered to be reservoirs of vaccine-induced plasma cells that support production and maintenance of circulating antibodies.

Together, these findings imply that the immunostimulating properties of BLPs rely mainly on the capacity to activate innate immune responses, or more specifically DCs, which are the crucial link between innate and specific adaptive responses. The capacity to induce maturation and activation of antigen-presenting cells (APCs) makes BLPs suitable for delivery of the associated antigen for presentation in the context of MHC class I and/or MHC class II.

### Involvement of TLR2

As demonstrated, the basis for successful induction of robust adaptive immune responses by BLP-based vaccines is activation of innate immune pathways. This is mainly mediated by non-specific recognition of invading pathogens, pathogen-derived components, or microbial-derived components in general. More specifically, the innate immune response is activated by recognition of common microbial associated molecular patterns (MAMPs) by a family of innate immune receptors, such as family of Toll-like receptors (TLRs) (47–49). As a result of recognition of MAMPs by TLRs, an activated state of the innate immune cells is induced. Activated innate immune cells are capable of shaping the adaptive B- and T-cell-mediated responses, depending on the information extracted from the activation signal (49).

Toll-like receptors are expressed on the surface or in the endosomal compartment of innate immune cells, DCs in particular, and their engagement typically induces cytokine and chemokine production, activation of DCs and subsequent priming of adaptive immune response (50–52). Examples of TLR ligands are: lipopeptides and peptidoglycan (TLR2), viral dsRNA (TLR3), LPS (TLR4), flagelin (TLR5), viral ssRNA (TLR7), bacterial, or CpG DNA (TLR9). Activation of TLRs initiates a signaling cascade directed through the MyD88 and/or TRIF signaling adapters, which in turn activates various transcription factors (49). Due to their innate immune-stimulating properties, TLR ligands have been explored as promising new-generation adjuvants.

An advantage of this new generation of vaccine adjuvants is that their mode of action is well-understood and characterized. The first TLR ligand explored as an adjuvant in a registered vaccine formulation is the TLR4 ligand MPLA used in GSK’s HBV vaccine Fendrix^®^ and HPV vaccine Cervarix^®^. Moreover, it has been shown that injection of healthy human subjects with CpG 7909 induces systemic innate immune activation manifested by expression of Th1-polarizing cytokines and IFN-inducible chemokines (53).

Bacterium-like particles belong to this group of new-generation immunostimulators, as its mode of action is well-defined and is based on activation of innate receptor – TLR2, which is a membrane surface receptor, specific for numerous bacterial, fungal, and viral components. Some of the ligands for TLR2 include: Zymosan (*S. cerevisiae*) (54), LPG (*L. major*) (55), LPP (56), and HSV (57). Recognition of TLR2 ligand by the receptor initiates the internalization of bound molecules by the endosome/phagosome system and leads to cellular activation. Consequently, cells of the innate immune system, e.g., DCs and macrophages, acquire functions of non-specific immune defense. The most important cytokines participating in this process of activation are TNF-α, IL-1α, IL-1β, IL-6, IL-8, and IL-12. TLR2 is expressed on a broad spectrum of different cells, such as monocytes, macrophages, DCs, microglia, polymorphonuclear leukocytes, and B- and T- cells. It is also expressed on the surface of airway epithelia, pulmonary alveoli, and skin keratinocytes (58–61). Very often, TLR2 functions in the form of a heterodimer, when it is associated with TLR1 or TLR6 (62).

The evidence that BLPs activate TLR2 was obtained from both *in vitro* (using TLR2-specific cell assays) and *in vivo* studies (immunization studies on TLR2−/− mice). For the *in vitro* studies, HEK293T cells expressing human TLR2, 3, 4, 5, 7, 8, and 9 and mouse TLR7 and 9, were stimulated with BLPs. The results showed that only the cell line expressing TLR2 responded to BLP stimulation. This observation was corroborated by the fact that the human DCs incubated with an anti-TLR2 antibodies showed a significant decrease in IL-6 production upon incubation with BLPs. These findings suggest that BLPs shape the immune response by activating TLR2 on innate immune cells, e.g., DCs (15). The precise mechanism of the TLR2 activation by BLPs is not completely understood and remains to be elucidated.

In addition to the indirect activation of adaptive responses through the interaction with DCs, recent literature suggests that TLR2 agonists also have a direct effect on the adaptive immune system by interacting with TLR2 expressed on the surface of T-cells (63). One of the consequences of this direct stimulation is induction of Th1-polarized effector responses (63). Furthermore, it has been shown that TLR2 ligands can interact directly with TLR2 on the surface of B-cells. Consequently, the MyD88 signaling cascade within B-cells is initiated, which can trigger IFNγ production by T-cells and T-cell dependent IgG2c/a antibody switch (64). Therefore, BLPs possibly have an additional mode of action by inducing Th1-polarized immune responses through direct interaction with TLR2 present on T- and B-cells.

In addition to the described *in vitro* studies, intranasal immunizations of wild type (*wt*) and TLR2 knockout mice (TLR2−/−) were performed to determine the *in vivo* effects of the absence of TLR2. This study confirmed that nasal vaccination of mice with influenza split-virion vaccine mixed with BLPs (FluGEM-A) induces local and systemic T- and B-cell responses in a TLR2-dependent manner (Keijzer, personal communication). More specifically, upon vaccination with FluGEM-A vaccine the number of IFNγ-producing cells in the local draining lymph nodes and spleen was significantly reduced in TLR2−/− mice compared to *wt* mice. Interestingly, absence of TLR2 in knockout mice only moderately effected the IgG levels after vaccination. However, the results of the study showed that class switch toward IgG2c antibody was closely dependent on the interaction of BLP with TLR2, while the levels of IgG1 were very similar between the groups. In line with the previously mentioned study (64), the lack of antibody switch toward IgG2c is closely related to and can be explained by the substantial decrease in IFNγ production in TLR2−/− mice, as this cytokine plays a major role in potentiating IgG2a/c antibody isotype switch. Finally, lack of TLR2 signaling had a negative impact on the development of local mucosal responses, as S-IgA titers measured in nasal and vaginal lavages of TLR2−/− mice vaccinated with FluGEM-A were largely absent (Table 3).

**Table 3:** **Immunostimulatory capacity of BLPs *in vivo* critically depends on TLR2 activation**.

Mousetype	IFNγ-producing cells(per 10^6^ cells)		IAV-specific B-cells(per 10^6^ cells)		Serum IgG(μg/ml)	Serum IgG2c(μg/ml)	Serum IgG1(μg/ml)	S-IgA titer	
	LN	Spleen	LN	Spleen				Nasal lavage	Vaginal lavage
**BLP-INDUCED IMMUNE RESPONSE CRITICALLY DEPENDS ON TLR2 ACTIVATION**									
TLR2−/−	30	102	3	4	10.5	1.0	8.0	–	0.5
*wt*	98	2701	10	8	24.4	6.3	4.9	2.5	7.1

The summarized results provide evidence that the mucosal immunostimulating activity of BLPs, in terms of robust activation of both systemic and local immune responses, depends critically on activation through (innate) TLR2 activation by BLPs.

## Safety and Immunogenicity of BLP-Based Vaccines in Humans: FluGEM-A Phase I Clinical Trial

The safety, tolerability, reactogenicity (primary end-point), and immunogenicity (secondary end-point) of intranasal FluGEM-A vaccine composed of seasonal trivalent inactivated influenza vaccine (TIV, season 2009–2010) mixed with BLPs, was evaluated in a randomized, double-blind, controlled phase I clinical trial in male and female subjects aged between 18 and 49 years of age. As a parallel control vaccine, i.n. administered non-adjuvanted TIV antigen was used. Details of the study are prepared for publication elsewhere. Primary safety end-points for FluGEM-A were not distinguishable from those of plain unadjuvanted TIV with respect to severity, duration, and number of adverse events (AEs). No severe AEs were reported. Moreover, there was no evidence of increased frequency of complaints after the second administration, suggesting no cumulative effect of vaccination with intranasal BLP-based vaccine. Based on the data summarized above, we conclude that intranasal FluGEM-A vaccine tested in human adults is safe and well tolerated as far as can be determined in a limited number of study subjects. As an exploratory safety parameter the anti-lactococcal serum antibody response before and after complete vaccination was measured in order to determine the suitability of repeated use of BLP-based vaccines in humans. In addition, we summarize below results of primary and exploratory immunology analyses, obtained for the FluGEM-A group vaccinated with 1.25 mg BLPs mixed with a standard TIV antigen dose of 15 μg HA per strain and the TIV antigen only group with the same standard HA dose per strain as the FluGEM-A group. The two study groups consisted of 15 subjects each. The vaccine dose was administered in a volume of 250 μl, equally divided between two nostrils. Seasonal TIV was composed of the following viral strains: A/California/7/2009 (H1N1), A/Perth/16/2009 (H3N2), and B/Brisbane/60/2008. The subjects received on day 0 and 21 a single intranasal dose of FluGEM-A or TIV alone and were followed for 210 days post-vaccination. The primary analysis of systemic immunogenicity was through systemic HI titers against each of the included viral strains and mucosal (nasal) influenza-specific S-IgA antibody levels. In addition, for exploratory purposes, antigen-specific IFNγ-producing cells were enumerated among peripheral blood mononuclear cells (PBMCs) collected on days 0, 7, 21, and 28 post-prime vaccination.

### FluGEM-A vaccine is suitable for repeated vaccination: Response against the BLP carrier

Another important aspect of BLP-based vaccines is whether antibodies against the *L. lactis*-derived immune-stimulating component, are induced. These may possibly hamper (repeated) vaccination with BLP-based vaccines. For this purpose, the status of *L. lactis*-specific antibodies prior- and post-vaccination was determined.

**Figure 6:**
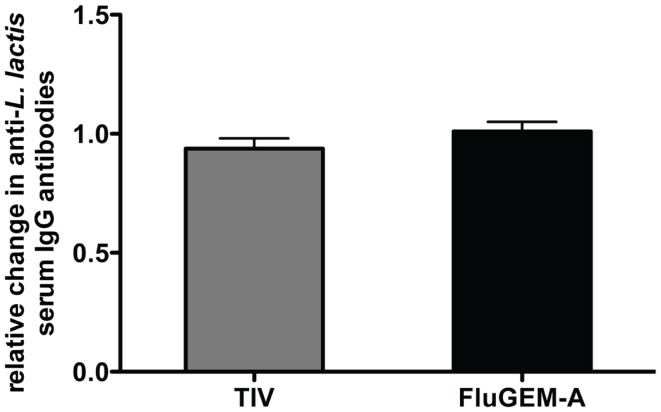
**Relative change in BLP-specific antibody titer after i.n. vaccination with FluGEM-A**. Blood samples were collected from all test subjects and *L. lactis*-specific antibody titers were determined on study days 0 (baseline titers) and 42. In both vaccination groups (i.m. TIV and i.n. FluGEM-A) no increase in *L. lactis*-specific antibody titers due to vaccination were measured.

In all the subjects, serum antibody titers against *L. lactis* proteins were measured before the start of the vaccination (day 0) and 3 weeks after the second vaccination (day 42). Pre-vaccination levels of *L. lactis*-specific antibodies in all the subjects were high (in the FluGEM-A group the mean pre-vaccination titer was 1.3 × 10^4^, with max = 4.2 × 10^4^ and min = 2.3 × 10^3^; in the TIV-only group the mean pre-vaccination titer was 1.3 × 10^4^, with max = 5.1 × 10^4^ and min = 0.8 × 10^3^). Figure 6 represents the relative change in *L. lactis*-specific antibody titers 3 weeks after the final vaccination showing that vaccination with FluGEM-A did not increase the level of *L. lactis*-specific antibodies. In both vaccination groups no changes in antibody titers were measured. Thus, the results indicate that BLPs do not enhance the levels of *L. lactis*-specific antibodies, which suggests FluGEM-A and other BLP-based vaccines are suitable for repeated administration.

### Immunogenicity of intranasally delivered FluGEM-A vaccine

The primary measured immunological parameters were serum HI titers and mucosal (nasal) influenza-specific S-IgA against each of the viral strains included in the vaccine. Additionally, in order to characterize cellular immune responses upon vaccination, PBMCs were tested for presence of IFNγ-secreting cells.

#### Hemagglutination inhibition titers

Serum HI titers represent the generally accepted surrogate marker for evaluation of efficacy of influenza vaccines and vaccination protocols. Table 4 summarizes results of HI geometric mean titers (GMTs) and GMT ratios measured in all the study subjects on day 0, 21, 42, and 210, regardless of their baseline HI titers, against all the influenza strains included in the vaccine. A clear increase in HI titers against all strains present in the vaccine was already observed after one i.n. administration with FluGEM-A. No additional increase in titers was observed after administration of the second vaccine dose. Although the titers observed after FluGEM-A administration were higher for all strains at each time-point, the HI levels obtained after administration with TIV-only were already surprisingly high. As shown in Table 4, i.n. vaccination with FluGEM-A induced GMT ratios >2.5, which were at least twice as high as the ratios recorded in TIV-only group. Importantly, HI GMT titers ≥40 were achieved in subjects i.n. vaccinated with FluGEM-A after administration of only one vaccine dose. The titers against all three strains remained stable throughout the whole follow-up period of almost 6 months, which is in line with previously presented results of duration of immunity induced by vaccination of mice with FluGEM-A.

**Table 4:** **HI titers specific for all three influenza strains included in the vaccine**.

Vaccination group	TIV				FluGEM-A			
**HI GEOMETRIC MEAN TITERS AND RATIO TO BASELINE TITERS**								
Study day	0	21	42	210	0	21	42	210
**Influenza B strain**
Number of subjects	13	13	13	13	10	10	10	10
HI GMT	10.5	29.9	32.4	50.6	8.5	48.1	47.8	63.9
HI GMT ratio	1.0	2.8	3.1	4.8	1.0	5.7	5.6	7.6
**Influenza H1N1 strain**
Number of subjects	11	11	11	11	11	11	11	11
HI GMT	10.4	49.5	53.8	40.8	23.2	118.0	109.9	131.7
HI GMT ratio	1.0	4.8	5.2	3.9	1.0	5.1	4.7	5.7
**Influenza H3N2 strain**
Number of subjects	15	15	15	15	12	12	12	12
HI GMT	13.2	197.2	243.7	179.5	23.1	261.9	287.0	244.3
HI GMT ratio	1.0	14.9	18.5	13.6	1.0	11.3	12.4	10.6

HI titers were measured on study days 0 (baseline) 21, 42, and 210.

Intranasal FluGEM-A vaccine fulfilled the EMA criteria for a seasonal influenza vaccine, i.e., at least one criteria out of the following was met: seroconversion in >40% of subjects, seroprotection (HI titers ≥40) in >70% of subjects, and mean geometric increase in titer >2.5, for each influenza strain included in the vaccine.

**Figure 7:**
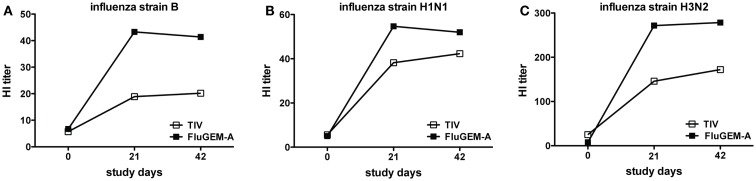
**Change in HI titers against (A) influenza B, (B) influenza H1N1 (C) and influenza H3N2 upon vaccination**. HI titers in sera of study subjects were measured on study days 0 (baseline titers), 21, and 42. Only titers measured in subjects with baseline <40 are depicted. For all three influenza strains, responses induced by i.n. vaccination with FluGEM-A were faster and of higher-magnitude, when compared to responses induced by i.n. vaccination with TIV (barely reach protective titer of 40).

To describe the dynamics in HI titers more accurately, in Figure 7 we depict the change in HI titers for all three influenza strains after vaccination, measured in subjects with HI baseline titers <10. For all three influenza strains, in particular B and H3N2, vaccination with FluGEM-A induced higher-magnitude responses, when compared to responses induced by i.n. vaccination with TIV-only vaccine. Additionally, GMTs induced upon i.n. vaccination with control TIV vaccine did not (strain B) or barely (strain H1N1) reach the protective threshold titer of 40.

#### Influenza-specific mucosal (nasal) IgA response

**Figure 8:**
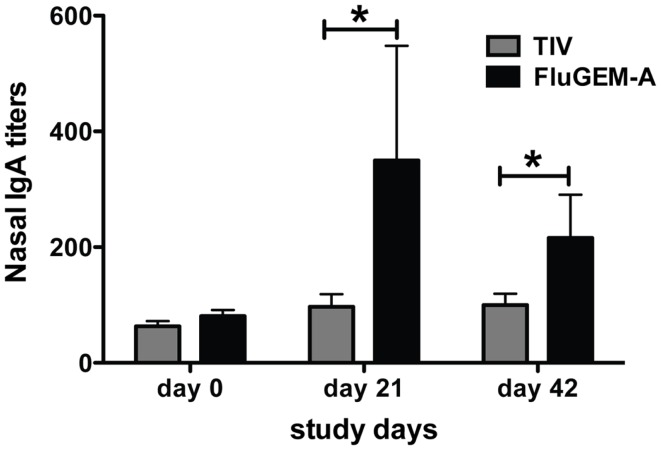
**Influenza-specific IgA titers measured in nasal lavages of vaccinated subjects**. Nasal lavages of study subjects were collected and IgA titers assessed on study days 0 (baseline), 21, and 42. On both days 21 and 42 titers measured in nasal lavages of subjects vaccinated i.n. with FluGEM-A were significantly higher than titers measured in lavages of subjects vaccinated i.n. with TIV. Increase in nasal IgA titers in FluGEM-A vaccination group relative to the baseline was approximately 100% by day 21. *p < 0.05; one-tailed Mann–Whitney *U* test.

To evaluate the capacity of FluGEM-A to induce local mucosal responses, influenza-specific S-IgA titers in nasal washes of vaccinated subjects were measured. Figure 8 summarizes the titers of the nasal influenza-specific S-IgA antibodies. Only subjects with S-IgA values available at all the time points are included in the table.

An increase of approximately fourfold relative to the baseline titers was recorded in the FluGEM-A group already on day 21. This was associated with a high seroconversion rate of approximately 70%. In contrast, only a minor increase in influenza-specific S-IgA levels was detected in nasal washes of some of the participants in the control TIV group after vaccination. Thus, i.n. vaccination with FluGEM-A induces, in addition to systemic response (HI titers), a robust nasal S-IgA response.

#### Cellular (IFNγ) response

**Figure 9:**
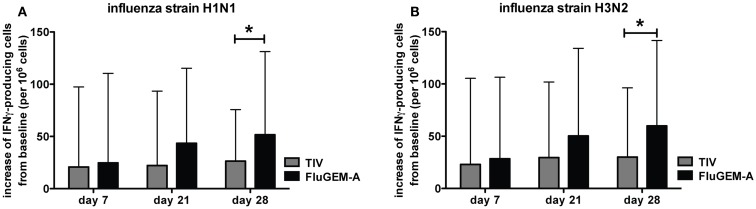
**FluGEM-A induced increase in influenza H1N1- (A) and H3N2-specific (B) IFNγ-producing T-cells**. Blood samples were collected from study participants on study days 0, 7, 21, and 28. PBMCs were isolated and IFNγ-producing cells were enumerated by ELISPOT assay. Results are presented as a mean increase from a baseline (number of specific cells measured on day 0 per 10^6^ cells) with 95% confidence interval depicted. *p < 0.05; two-tailed Student *t-*test.

IFNγ is a cytokine whose immunostimulatory and immunomodulatory functions are critical for both innate and adaptive immune responses. To evaluate the IFNγ responses induced by vaccination with FluGEM-A, PBMCs were collected from vaccinated subjects on days 0, 7, 21, and 28 and the number of IFNγ-producing cells were enumerated after restimulation with TIV. Figure 9 represents the vaccination-induced increase in number of IFNγ-producing cells, specific for H1N1 (Figure 9A) and H3N2 (Figure 9B) influenza strains. Results are presented as a mean increase from baseline (number of specific cells measured on day 0 per 10^6^ cells) with 95% confidence intervals depicted. Presented results show a trend of increase in the number of specific IFNγ-producing cells from day 0 to day 21 among PBMCs isolated from subjects vaccinated i.n. with FluGEM-A and the increase became significant for both strains after the booster immunization. This trend and significant increase at day 28 is not observed in PBMCs isolated from subjects vaccinated i.n. with TIV and a plateau in the number of IFNγ producing PBMCs in this vaccination group is reached 1 week after the primary vaccination.

We conclude that i.n. vaccination with FluGEM-A, in addition to activation of humoral immune response, and unlike i.n. vaccination with TIV, stimulates cellular responses in human subjects, as shown by a vaccine-induced increase in the number of influenza-specific IFNγ-producing PBMCs.

## Final Remarks

Mucosal vaccines offer several advantages, as demonstrated by the described BLP-based vaccines, in comparison to the current, classical, injectable vaccines. Besides the ease of needle-free administration, mucosal vaccines are capable of inducing both systemic and local responses at the surface of mucosae. In this way, these vaccines already provide a first line of defense in the form of S-IgA at the port of entry of most pathogens. Additionally, locally produced S-IgA antibodies have been suggested to be less sensitive to antigenic variation (28) and may therefore provide broader protection. Our observations with FluGEM-A in heterologous challenge studies seem to be in line with this hypothesis (12).

The limited immunogenicity data derived from the Phase I clinical trial appears to confirm the promising preclinical data. Subjects that received FluGEM-A via the i.n. route showed systemic HI titers and local S-IgA responses. Induction of humoral responses was associated with the production of the immunomodulatory cytokine, IFNγ. Whether the local and systemic responses induced in humans by mucosal vaccination with FluGEM-A in particular and BLP-based vaccines in general will result in improved protection against influenza and other pathogen infections, respectively, now remains to be investigated in follow-up trials.

Development of a new generation of mucosal adjuvants and immunostimulatory platforms, of which BLPs are an example, fuels the required evolution in vaccine design and protection against pathogen infections. Well-understood modes of action of such compounds allows for rational design of safe and protective vaccines with the desired immune responses.

## Conflict of Interest Statement

The authors are employees of the organization that funded all the work described in the paper.
